# Supplementary material for: Transitions between cognitive topographies: contributions of network structure, neuromodulation, and disease
Source: bioRxiv. 2023 Mar 17:2023.03.16.532981. Preprint. [Version 1] doi: 10.1101/2023.03.16.532981 (PMC10055141; doi:10.1101/2023.03.16.532981)
Supplement: Supplementary file 1 [file NIHPP2023.03.16.532981v1-supplement-1.pdf]

bioRxiv preprint doi: https://doi.org/10.1101/2023.03.16.532981; this version posted March 17, 2023. The copyright holder for this preprint (which was not certified by peer review) is the author/funder, who has granted bioRxiv a license to display the preprint in perpetuity. It is made available under aCC-BY 4.0 International license.

Supplementary Materials

bioRxiv preprint doi: https://doi.org/10.1101/2023.03.16.532981; this version posted March 17, 2023. The copyright holder for this preprint (which was not certified by peer review) is the author/funder, who has granted bioRxiv a license to display the preprint in perpetuity. It is made available under aCC-BY 4.0 International license.

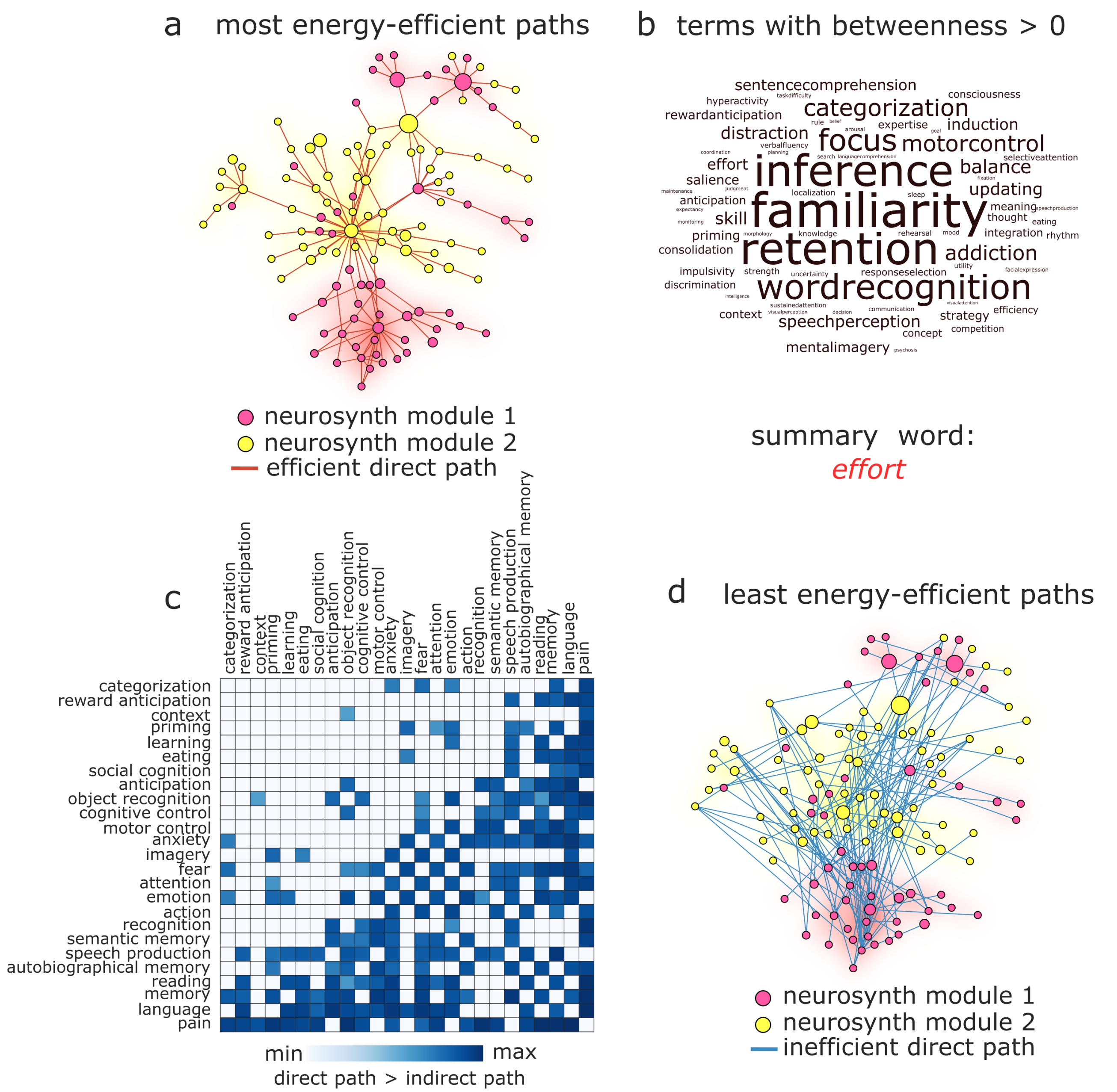

Figure S1. **Efficient and inefficient paths between cognitive topographies |** **(a)** Representation of the control energy matrix as a weighted network, showing the transitions (edges) between cognitive topographies (nodes) that require the least energy (for display purposes, only 10% of connections are shown). Nodes are colored according to their membership of the two communities into which cognitive topographies cluster. Cognitive topographies that act as intermediaries for the most efficient transition between two other cognitive topographies correspond to nodes that have non-zero betweenness centrality. Node size reflects the betweenness centrality of each node (taking into account all paths). **(b)** The NeuroSynth terms corresponding to cognitive topographies with non-zero betweenness centrality in the network representation in (a); size reflects the betweenness centrality of the corresponding nodes. To identify the term that most summarises all others, we represent each term as a high-dimensional vector in semantic space using *word2vec* [94], and we measure their similarity using cosine similarity between these vector representations. We find that "effort" has the highest mean cosine similarity with the vector representations of all other high-betweenness terms. **(c)** Matrix of the transition cost between each pair of cognitive topographies in the reduced set, showing the difference in control energy between the direct and least-expensive paths; the value of each non-empty cell indicates the energy premium incurred by taking the direct path between two cognitive topographies. **(d)** The most costly direct paths between cognitive topographies (only 10% shown, for display purposes). Node size, colour, and position are the same as in (a).

bioRxiv preprint doi: https://doi.org/10.1101/2023.03.16.532981; this version posted March 17, 2023. The copyright holder for this preprint (which was not certified by peer review) is the author/funder, who has granted bioRxiv a license to display the preprint in perpetuity. It is made available under aCC-BY 4.0 International license.

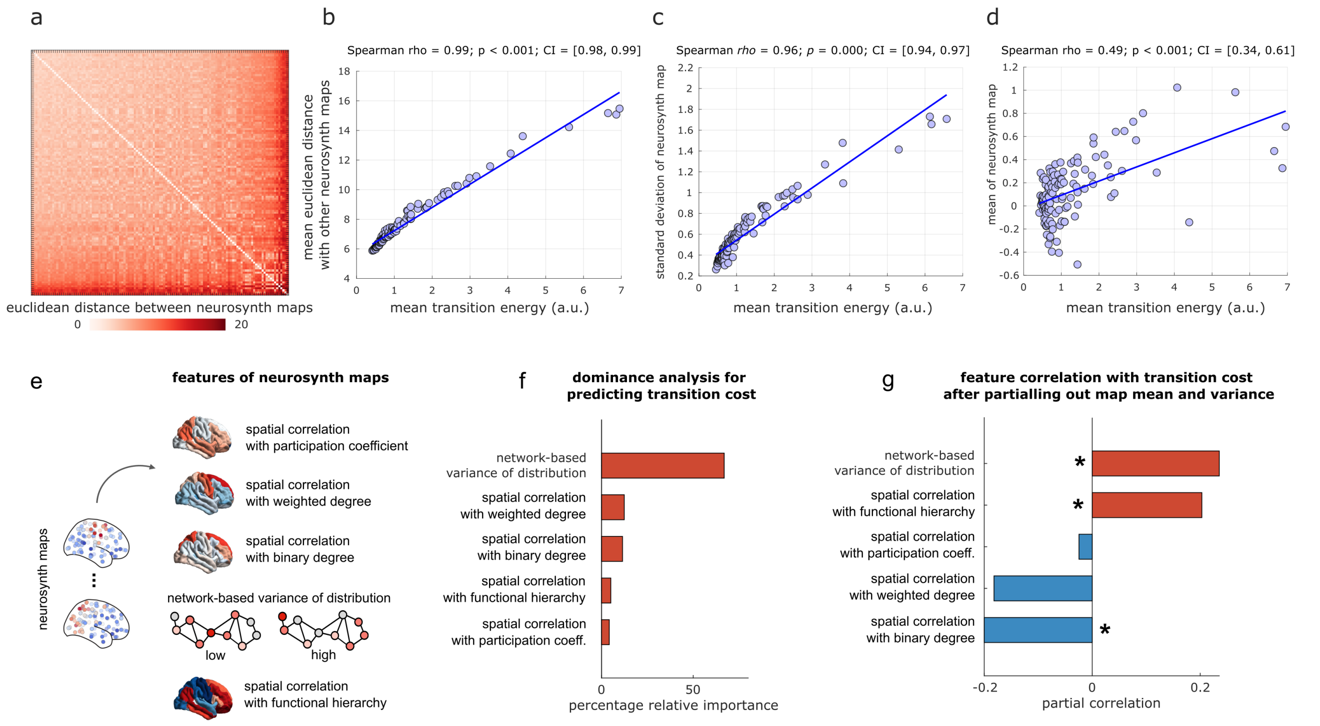

Figure S2. **Predictors of transition energy.** **(a)** Euclidean distance between the vectors corresponding to each NeuroSynth map. **(b)** Transition energy to a given cognitive topography (averaged over all starting states) correlates with the mean Euclidean distance between its corresponding NeuroSynth map and all others. **(c)** Transition energy to a given cognitive topography (averaged over all starting states) correlates with the standard deviation of its NeuroSynth map. **(d)** Transition energy to a given cognitive topography (averaged over all starting states) correlates with the mean of its NeuroSynth map. **(e)** Mean and variance of a NeuroSynth map are coarse descriptions that do not account for neuroanatomy. As neuroanatomically-grounded predictors of transition energy, we consider the following features of each NeuroSynth map: (i) the map's spatial alignment with the regional distribution of participation coefficients, treating the structural connectome as a network; (ii) the map's spatial alignment with the regional distribution of weighted node degree, treating the structural connectome as a network; (iii) the map's spatial alignment with the regional distribution of binary node degree, treating the structural connectome as a network; (iv) a recently developed measure of variance for distributions over a network [28, 29]: unlike the usual measure of variance, which assumes independent data-points and is agnostic to their spatial location, this measure takes into account the relationships between observations. Specifically, variance of a distribution over a network is low, if high values occur at nodes that are easy to reach using a diffusion process along network paths of all length. Conversely, if the majority of high values occurs at nodes that are difficult to reach using diffusion, then the distribution will have high network-based variance. This is especially relevant because network control theory operationalises control inputs as spreading by diffusing over the network. (v) The map's spatial alignment with the principal gradient of functional connectivity (unimodal-transmodal hierarchy) [85]. **(f)** Bar plot shows the relative dominance of each predictor as obtained from dominance analysis [4]. Dominance analysis distributes the fit of the model across predictors such that the contribution of each predictor can be assessed and compared to other predictors, reflecting the proportion of the variance jointly explained by all predictors, that can be attributed to each predictor. **(g)** Bar plot shows the partial correlation between each predictor and the average cost to transition to a given brain state, after controlling for the effects of brain state mean and variance.

bioRxiv preprint doi: https://doi.org/10.1101/2023.03.16.532981; this version posted March 17, 2023. The copyright holder for this preprint (which was not certified by peer review) is the author/funder, who has granted bioRxiv a license to display the preprint in perpetuity. It is made available under aCC-BY 4.0 International license.

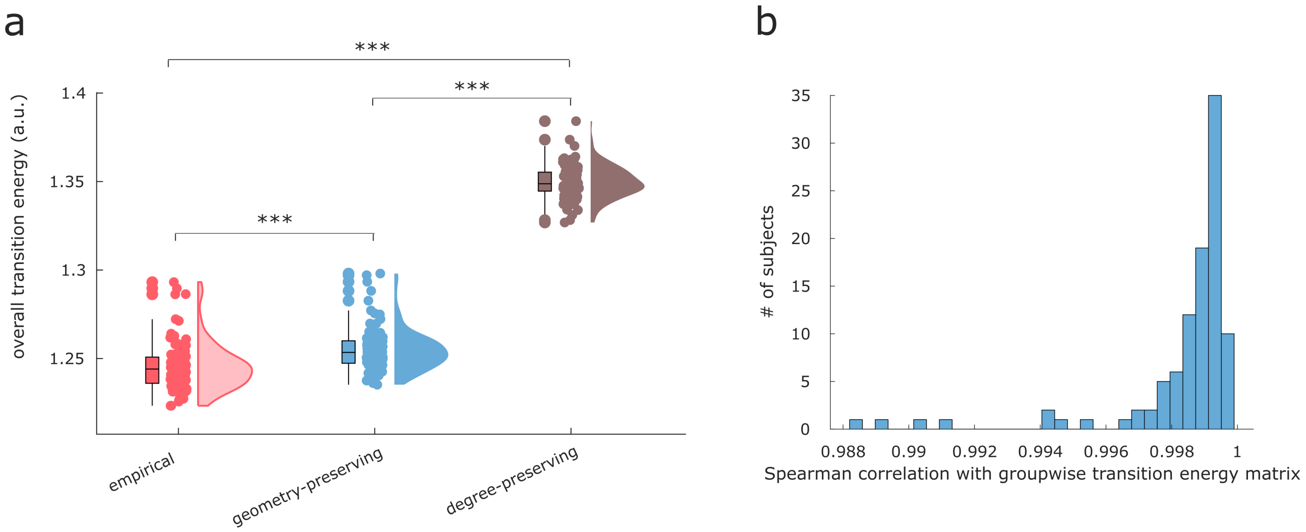

Figure S3. **Replication at the single-subject level. (a)** Overall transition energy (averaged across all transitions), for each subject and for the corresponding degree- and cost-preserving nulls. **(b)** Histogram of the correlation between each subject's matrix of transition energies, and the matrix of transition energies obtained from the group-wise consensus structural connectome.

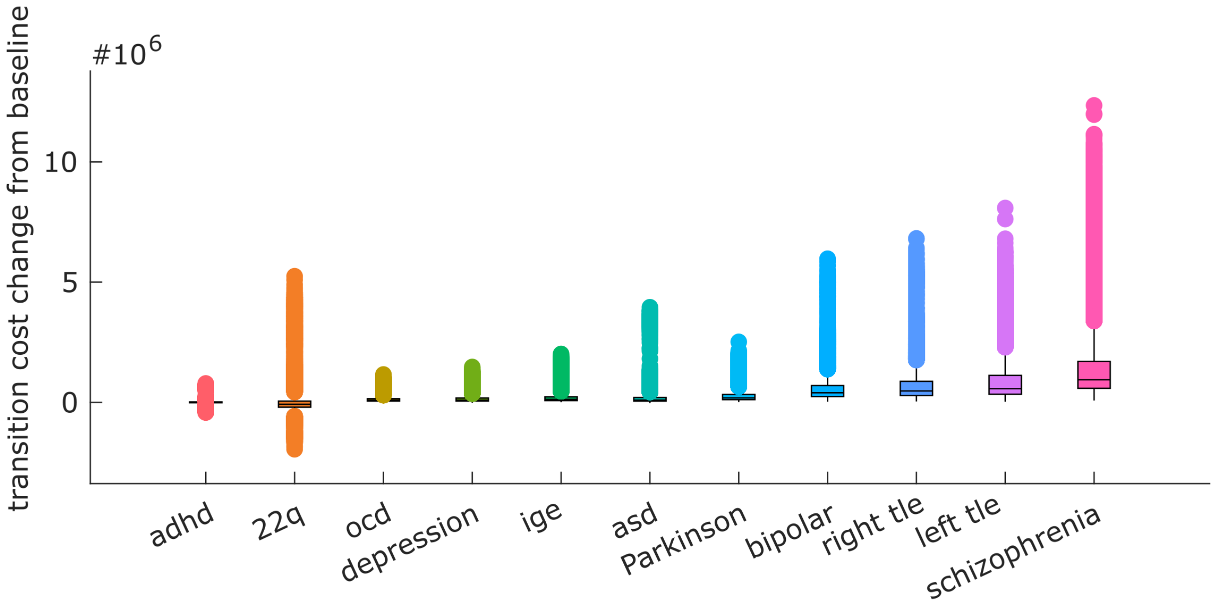

Figure S4. **Overall transition energy when applying control inputs according to cortical abnormality maps.** Each data-point represents the energy to transition to one target state, averaging across all source states.

bioRxiv preprint doi: https://doi.org/10.1101/2023.03.16.532981; this version posted March 17, 2023. The copyright holder for this preprint (which was not certified by peer review) is the author/funder, who has granted bioRxiv a license to display the preprint in perpetuity. It is made available under aCC-BY 4.0 International license.

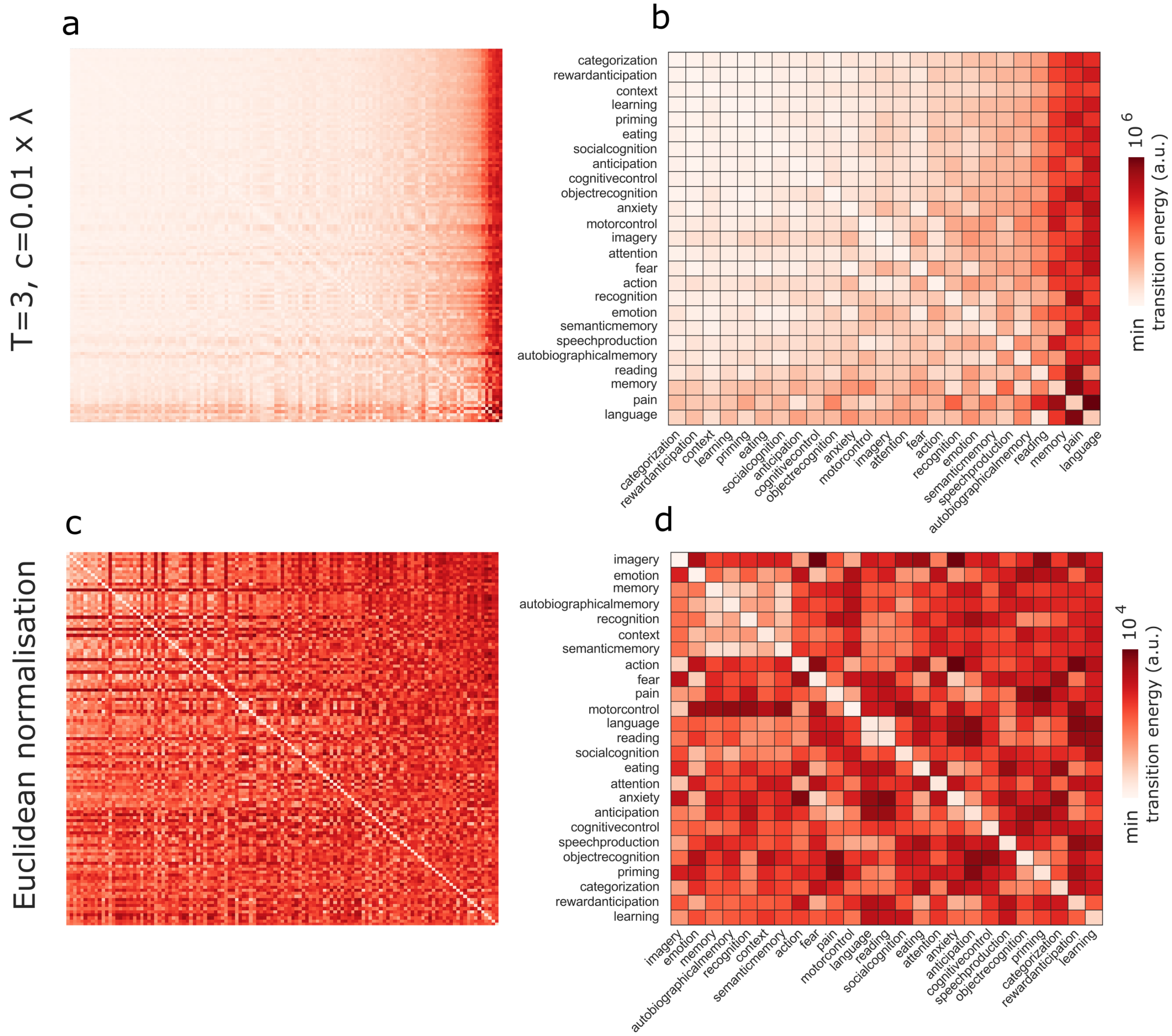

Figure S5. **Transition energies for alternative operationalisations of network control theory. (a,b)** Transition energy between each pair of 123 cognitive topographies from NeuroSynth (a) and the reduced set of 25 NeuroSynth terms (b), for network control with time horizon $T = 3$ and network normalisation factor $c = 0.01 \times |\lambda(A)_{\max}|$. Rows indicate source states, columns indicate target states. **(c,d)** Transition energy between each pair of 123 cognitive topographies from NeuroSynth (c) and the reduced set of 25 NeuroSynth terms (d), for network control with all NeuroSynth maps normalised to unit Euclidean norm.

bioRxiv preprint doi: https://doi.org/10.1101/2023.03.16.532981; this version posted March 17, 2023. The copyright holder for this preprint (which was not certified by peer review) is the author/funder, who has granted bioRxiv a license to display the preprint in perpetuity. It is made available under aCC-BY 4.0 International license.

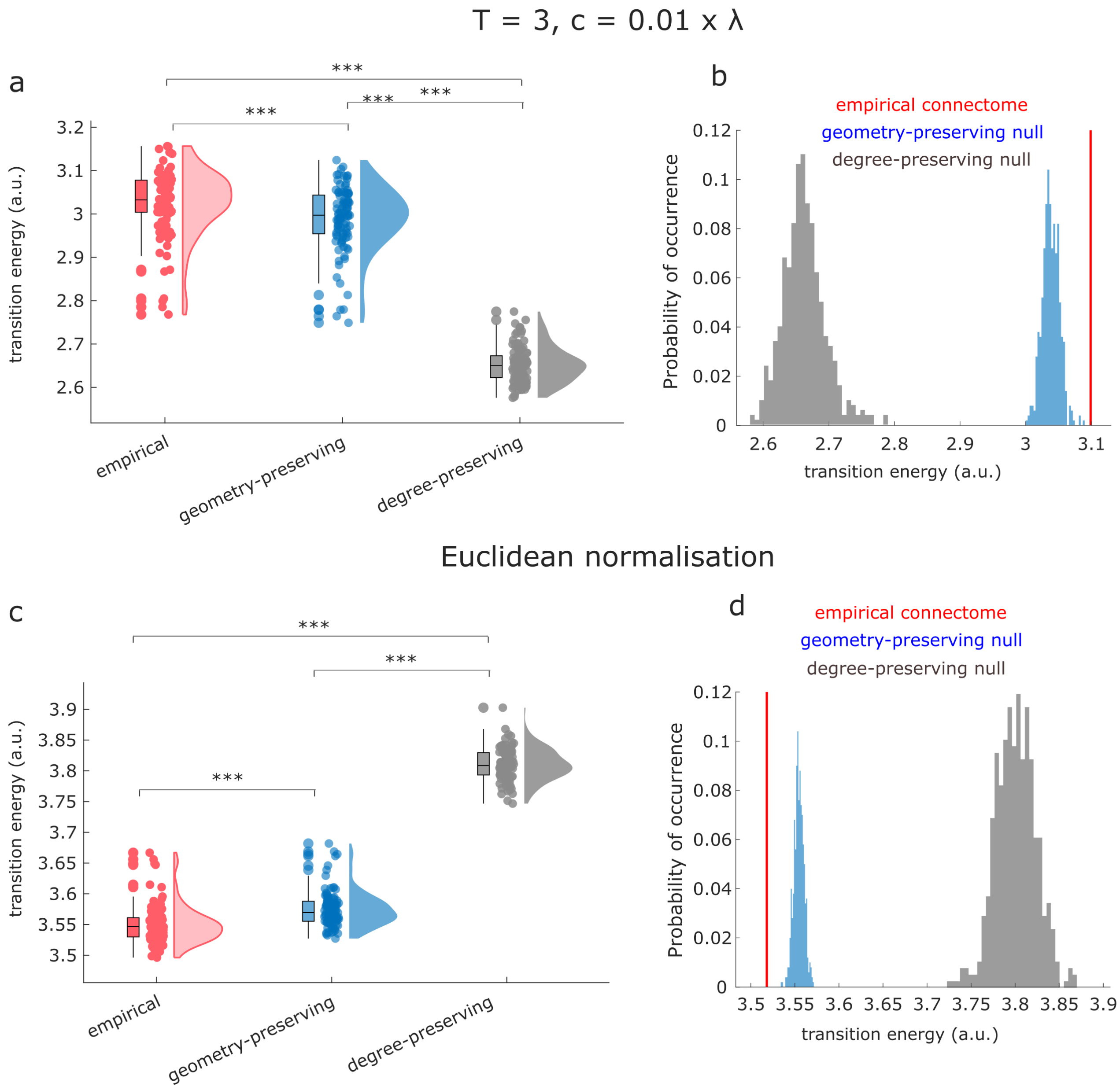

Figure S6. **Role of network topology in supporting transitions, for alternative operationalisations of network control theory. (a,b)** Subject-wise (a) and group-level (b) average transition energy for the empirical human connectome (red) and for degree-preserving (grey) and degree- and cost-preserving null models (blue), for network control with time horizon $T = 3$ and network normalisation factor $c = 0.01 \times |\lambda(A)_{\max}|$. **(c,d)** Subject-wise (c) and group-level (d) average transition energy for the empirical human connectome (red) and for degree-preserving (grey) and degree- and cost-preserving null models (blue), for network control with all NeuroSynth maps normalised to unit Euclidean norm.

bioRxiv preprint doi: https://doi.org/10.1101/2023.03.16.532981; this version posted March 17, 2023. The copyright holder for this preprint (which was not certified by peer review) is the author/funder, who has granted bioRxiv a license to display the preprint in perpetuity. It is made available under aCC-BY 4.0 International license.

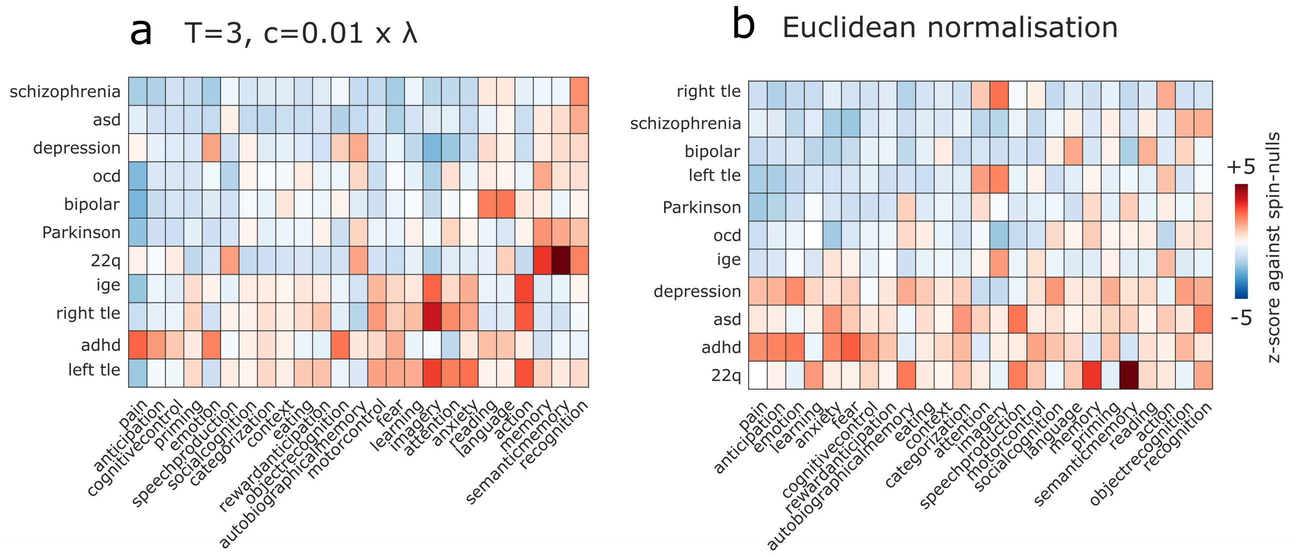

Figure S7. **Effects of disease-associated cortical atrophy, for alternative operationalisations of network control theory.** Heatmaps show how each disease reshapes the average transition energy required to reach a given cognitive topography from all other cognitive topographies, as a z-score against a null distribution of randomly rotated maps with preserved spatial autocorrelation and the same increases and decreases in cortical thickness, but occurring at different neuroanatomical locations. **(a)** For network control with time horizon $T = 3$ and network normalisation factor $c = 0.01 \times |\lambda(A)_{\max}|$; **(b)** for network control with all NeuroSynth maps normalised to unit Euclidean norm. adhd = attention deficit/hyperactivity disorder; asd = autistic spectrum disorder; ocd = obsessive-compulsive disorder; ige = idiopathic generalised epilepsy; right tle = right temporal lobe epilepsy; left tle = left temporal lobe epilepsy.

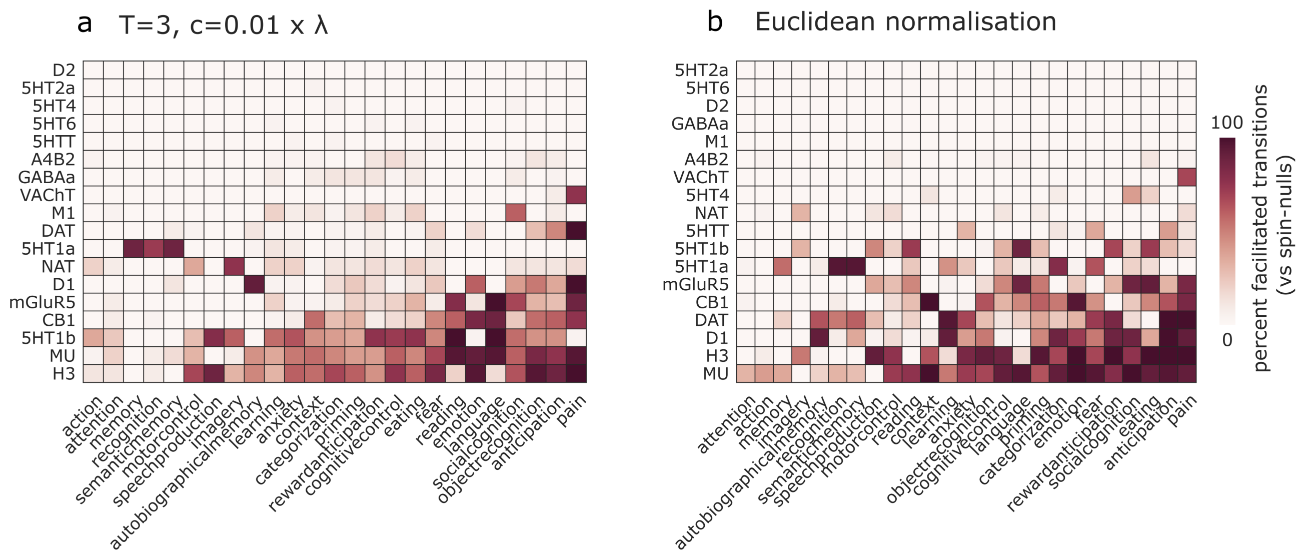

Figure S8. **How neurotransmitter systems can reshape the energy landscape of the human brain, for alternative operationalisations of network control theory.** Heatmaps show how each receptor/transporter reshapes the average cost of reaching a given cognitive brain state from all other states, as a percentage of transitions to each state that are facilitated, when compared against a null distribution of randomly rotated maps with preserved spatial autocorrelation and the same receptor/transporter density levels, but occurring at different neuroanatomical locations. **(a)** For network control with time horizon time horizon $T = 3$ and network normalisation factor $c = 0.01 \times |\lambda(A)_{\max}|$; **(b)** for network control with all NeuroSynth maps normalised to unit Euclidean norm.

bioRxiv preprint doi: https://doi.org/10.1101/2023.03.16.532981; this version posted March 17, 2023. The copyright holder for this preprint (which was not certified by peer review) is the author/funder, who has granted bioRxiv a license to display the preprint in perpetuity. It is made available under aCC-BY 4.0 International license.

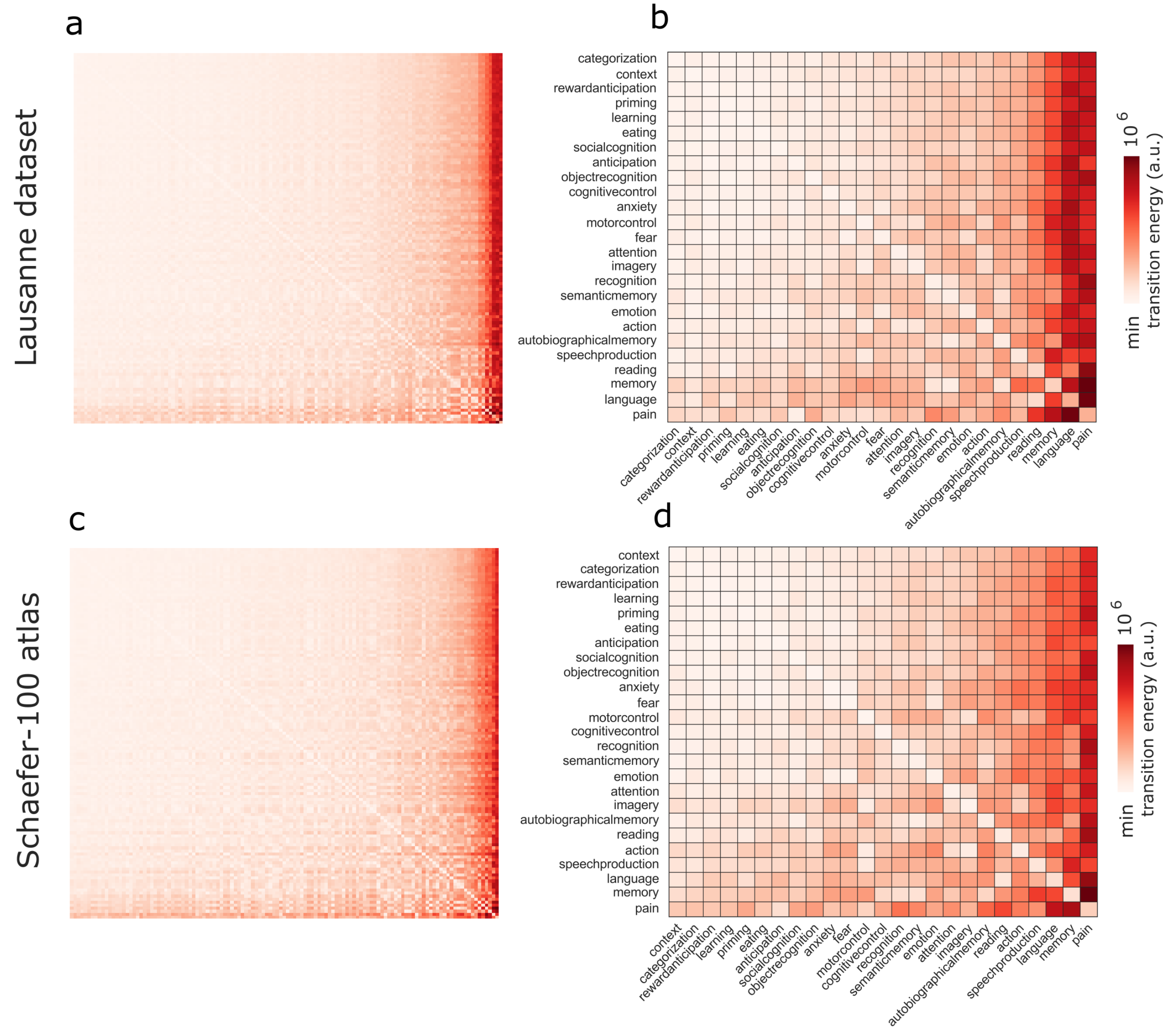

Figure S9. **Transition energies for alternative connectome dataset and alternative cortical parcellation. (a,b)** Transition energy between each pair of 123 cognitive topographies from NeuroSynth (a) and the reduced set of 25 NeuroSynth terms (b), for the Lausanne DSI dataset. Rows indicate source states, columns indicate target states. **(c,d)** Transition energy between each pair of 123 cognitive topographies from NeuroSynth (c) and the reduced set of 25 NeuroSynth terms (d), for Human Connectome Project data parcellated using the Schaefer-100 cortical atlas.

bioRxiv preprint doi: https://doi.org/10.1101/2023.03.16.532981; this version posted March 17, 2023. The copyright holder for this preprint (which was not certified by peer review) is the author/funder, who has granted bioRxiv a license to display the preprint in perpetuity. It is made available under aCC-BY 4.0 International license.

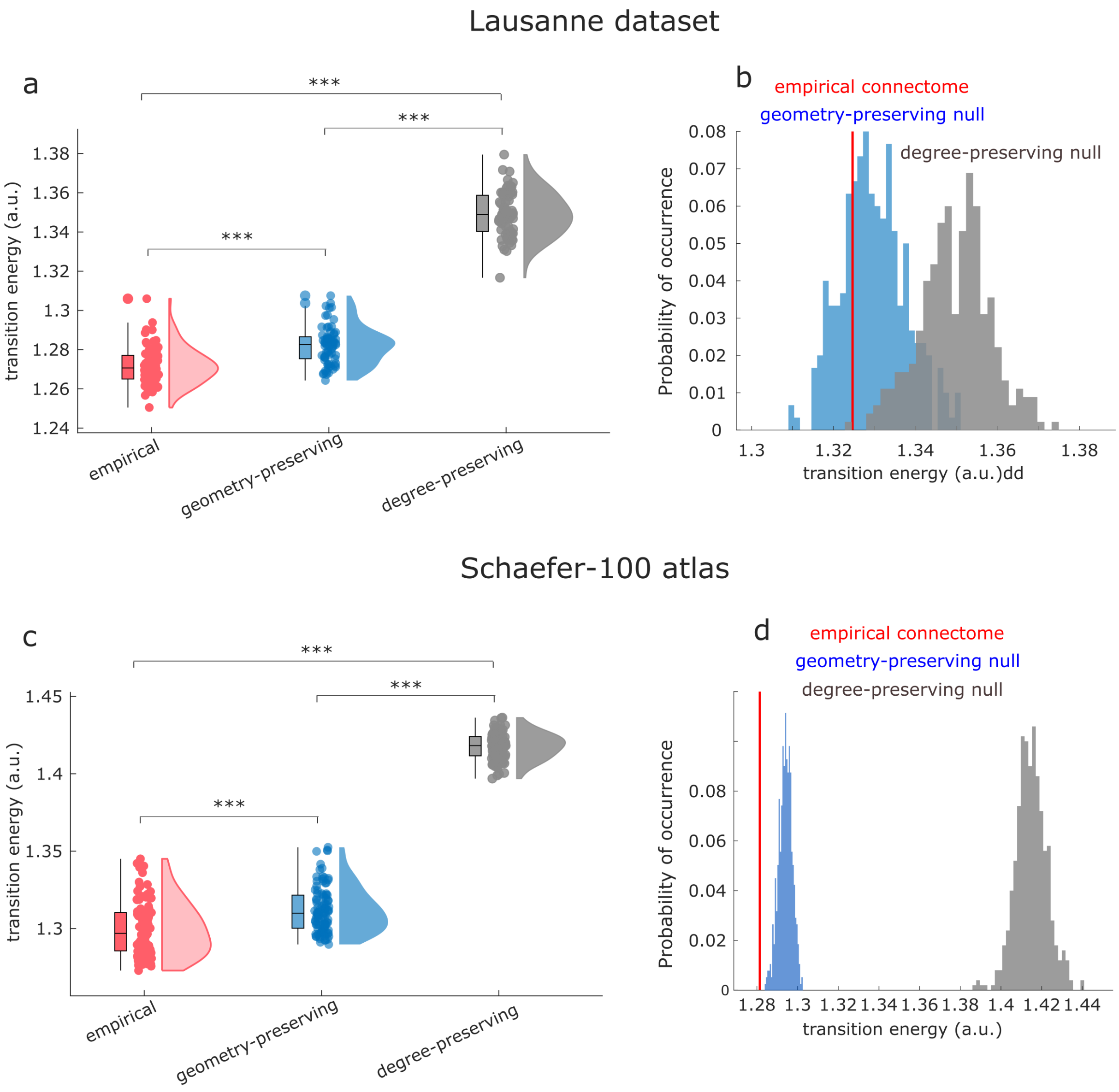

Figure S10. **Role of network topology in supporting transitions, for alternative connectome dataset and alternative cortical parcellation. (a,b)** Subject-wise (a) and group-level (b) average transition energy for the empirical human connectome (red) and for degree-preserving (grey) and degree- and cost-preserving null models (blue), for the Lausanne DSI dataset. **(c,d)** Subject-wise (c) and group-level (d) average transition energy for the empirical human connectome (red) and for degree-preserving (grey) and degree- and cost-preserving null models (blue), for Human Connectome Project data parcellated using the Schaefer-100 cortical atlas.

bioRxiv preprint doi: https://doi.org/10.1101/2023.03.16.532981; this version posted March 17, 2023. The copyright holder for this preprint (which was not certified by peer review) is the author/funder, who has granted bioRxiv a license to display the preprint in perpetuity. It is made available under aCC-BY 4.0 International license.

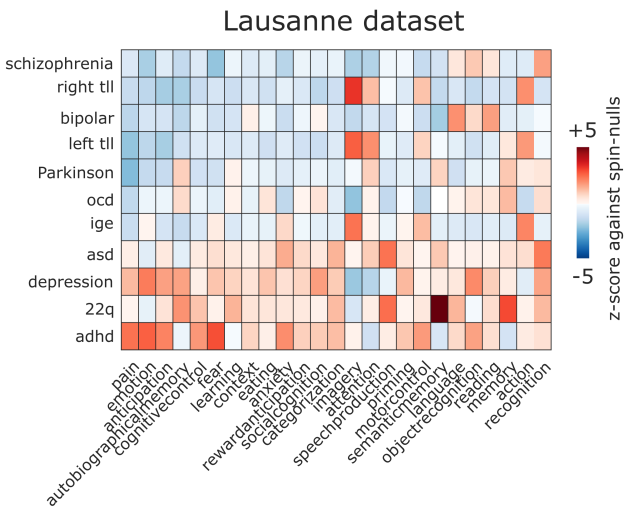

Figure S11. **Effects of disease-associated cortical atrophy, for alternative connectome dataset and alternative cortical parcellation.** Heatmaps show how each disease reshapes the average transition energy required to reach a given cognitive topography from all other cognitive topographies, as a z-score against a null distribution of randomly rotated maps with preserved spatial autocorrelation and the same increases and decreases in cortical thickness, but occurring at different neuroanatomical locations, for the Lausanne DSI dataset. adhd = attention deficit/hyperactivity disorder; asd = autistic spectrum disorder; ocd = obsessive-compulsive disorder; ige = idiopathic generalised epilepsy; right tle = right temporal lobe epilepsy; left tle = left temporal lobe epilepsy.

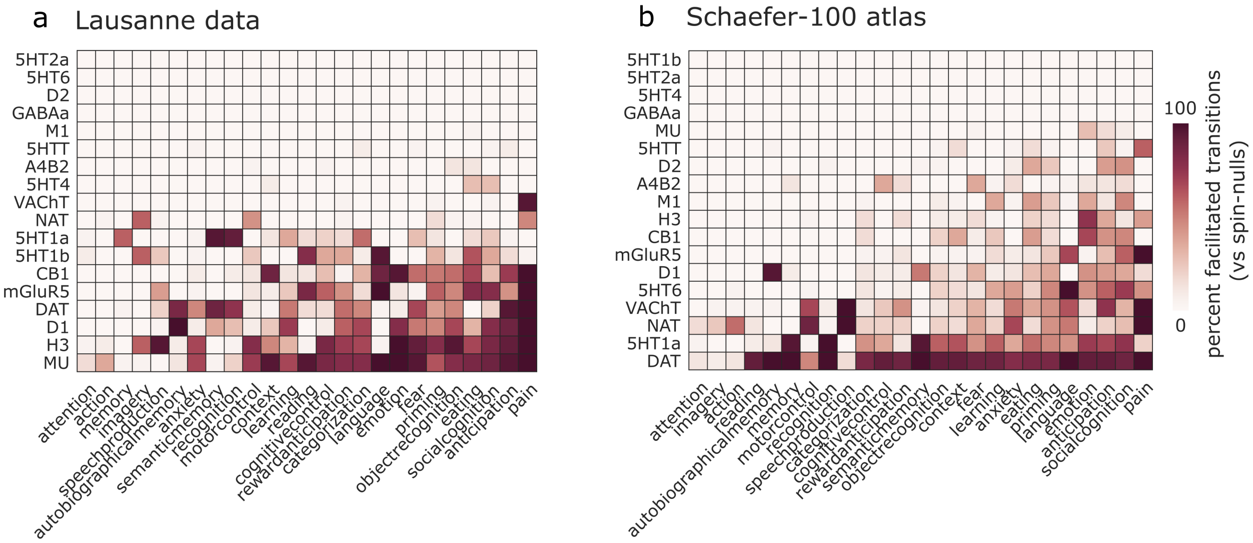

Figure S12. **How neurotransmitter systems can reshape the energy landscape of the human brain, for alternative connectome dataset and alternative cortical parcellation.** Heatmaps show how each receptor/transporter reshapes the average cost of reaching a given cognitive brain state from all other states, as a percentage of transitions to each state that are facilitated, when compared against a null distribution of randomly rotated maps with preserved spatial autocorrelation and the same receptor/transporter density levels, but occurring at different neuroanatomical locations. **(a)** For the Lausanne DSI dataset; **(b)** for Human Connectome Project data parcellated using the Schaefer-100 cortical atlas.

bioRxiv preprint doi: https://doi.org/10.1101/2023.03.16.532981; this version posted March 17, 2023. The copyright holder for this preprint (which was not certified by peer review) is the author/funder, who has granted bioRxiv a license to display the preprint in perpetuity. It is made available under aCC-BY 4.0 International license.

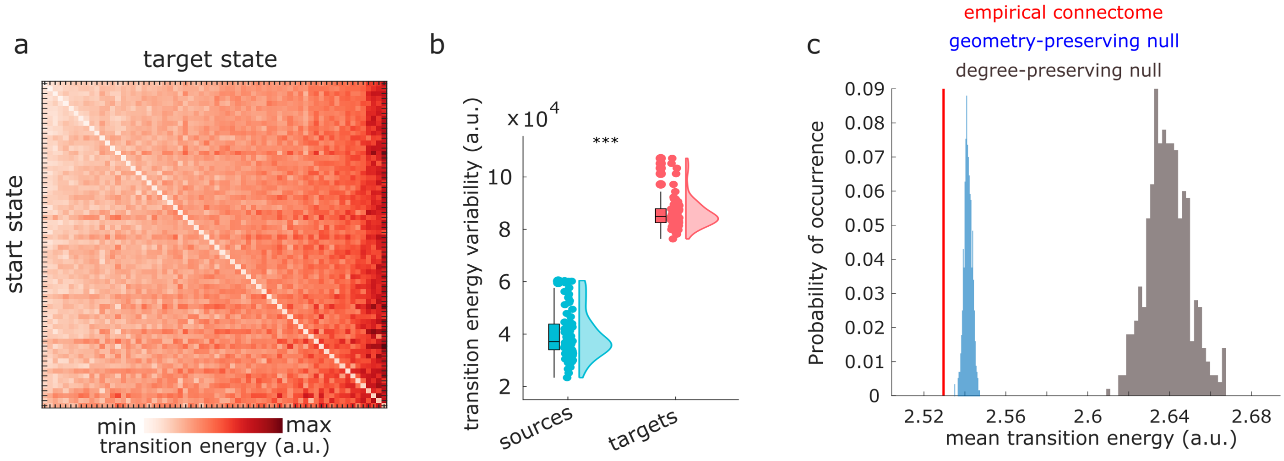

Figure S13. **Replication with cognitive topographies defined by BrainMap.** **a** Transition energy between each pair of 66 cognitive topographies from BrainMap. **b** Variability (standard deviation) of transition energy is greater along target states than along start states. **c** Degree-preserving randomised null models (grey) and null models that preserve the exact degree sequence and the approximate length distribution (blue) are significantly less favourable than the empirical human connectome (red) to support transitions between cognitive topographies defined by BrainMap.

bioRxiv preprint doi: https://doi.org/10.1101/2023.03.16.532981; this version posted March 17, 2023. The copyright holder for this preprint (which was not certified by peer review) is the author/funder, who has granted bioRxiv a license to display the preprint in perpetuity. It is made available under aCC-BY 4.0 International license.

	Mean1	SD1	Mean2	SD2	df	t-score	p-value	Effect Size
empirical vs geometry-preserving	1.25e+06	13400	1.26e+06	12200	99	-21.49	<0.001	-0.77
empirical vs degree-preserving	1.25e+06	13400	1.35e+06	9160	99	-60.18	<0.001	-9.05
degree- vs geometry-preserving	1.35e+06	9160	1.26e+06	12200	99	58.92	<0.001	8.7

TABLE S1. Subject-level comparison against null networks. Statistical comparison between subject-level overall transition energy distributions, for the empirical human connectome ($N = 100$ Human Connectome Project subjects) and corresponding degree-preserving and degree- and cost-preserving rewired nulls.

	Mean1	SD1	Mean2	SD2	df	t-score	p-value	Effect Size
empirical vs geometry-preserving	1.27e+06	9560	1.28e+06	9630	69	-15.03	<0.001	-1.1
empirical vs degree-preserving	1.27e+06	9560	1.35e+06	11300	69	-44.91	<0.001	-7.3
degree- vs geometry-preserving	1.35e+06	11300	1.28e+06	9630	69	36.34	<0.001	6.3

TABLE S2. Subject-level results for the Lausanne dataset. Statistical comparison between subject-level overall transition energy distributions, for the empirical human connectome ($N = 70$ subjects from the Lausanne dataset) and corresponding degree-preserving and degree- and cost-preserving rewired nulls.

	Mean1	SD1	Mean2	SD2	df	t-score	p-value	Effect Size
empirical vs geometry-preserving	1.3e+06	17200	1.31e+06	14900	99	-17.38	<0.001	-0.76
empirical vs degree-preserving	1.3e+06	17200	1.42e+06	8480	99	-64.11	<0.001	-8.6
degree- vs geometry-preserving	1.42e+06	8480	1.31e+06	14900	99	65.11	<0.001	8.7

TABLE S3. Subject-level results for Schaefer-parcellated data. Statistical comparison between subject-level overall transition energy distributions, for the empirical human connectome ($N = 100$ subjects from the Human Connectome Project) and corresponding degree-preserving and degree- and cost-preserving rewired nulls.

	Mean1	SD1	Mean2	SD2	df	t-score	p-value	Effect Size
empirical vs geometry-preserving	3.03e+05	7640	2.99e+05	7460	99	14.81	<0.001	0.49
empirical vs degree-preserving	3.03e+05	7640	2.65e+05	4140	99	50.37	<0.001	6.1
degree- vs geometry-preserving	2.65e+05	4140	2.99e+05	7460	99	-47.26	<0.001	-5.6

TABLE S4. Subject-level results for alternative implementation of network control theory. Statistical comparison between subject-level overall transition energy distributions, for the empirical human connectome ($N = 100$ HCP subjects) and corresponding degree-preserving and degree- and cost-preserving rewired nulls, for network control with time horizon $T = 3$ and network normalisation factor $c = 0.01 \times |\lambda(A)_{\text{max}}|$.

	Mean1	SD1	Mean2	SD2	df	t-score	p-value	Effect Size
empirical vs geometry-preserving	35500	312	35800	298	99	-27.19	<0.001	-0.84
empirical vs degree-preserving	35500	312	38100	260	99	-61.91	<0.001	-9.02
degree- vs geometry-preserving	38100	260	35800	298	99	58.64	<0.001	8.3

TABLE S5. Subject-level results for alternative implementation of network control theory. Statistical comparison between subject-level overall transition energy distributions, for the empirical human connectome ($N = 100$ HCP subjects) and corresponding degree-preserving and degree- and geometry-preserving rewired nulls, for network control with NeuroSynth maps normalised to unit Euclidean norm
